# CONVERSION FACTOR FROM DOSEMETER READING TO AIR KERMA FOR NUCLEAR WORKER USING ANTHROPOMORPHIC PHANTOM FOR FURTHER CONVERSION FROM AIR KERMA TO ORGAN-ABSORBED DOSE

**DOI:** 10.1093/rpd/ncaa051

**Published:** 2020-05-04

**Authors:** Hiroshige Furuta, Norio Tsujimura, Akemi Nishide, Shin’ichi Kudo, Shin Saigusa

**Affiliations:** 1 Institute of Radiation Epidemiology, Radiation Effects Association, 1-9-16 Kaji-cho, Chiyoda-ku, Tokyo 101-0044, Japan; 2 Nuclear Fuel Cycle Engineering Laboratories, Japan Atomic Energy Agency, Tokai-mura, Ibaraki 319-1194, Japan

## Abstract

Estimation of cancer risk based on the organ-absorbed dose is underway for the Japanese Epidemiological Study on Low-Dose Radiation Effects (J-EPISODE). The reconstruction method for the organ-absorbed dose follows the approach adopted in the IARC 15-Country Collaborative Study, which examined the dosemeter response to photon exposure for the old film badge (FB) type, a multi-element FB and a thermoluminescence dosemeter. Until 2000, the dosemeters used in Japan were almost the same in the IARC study, so IARC study data could be used as they were. However, since 2000, the type of dosemeter has been replaced with active personal dosemeters (hereafter called electronic personal dosemeters), radio-photoluminescent glass dosemeters (Glass badge) and optically stimulated luminescence dosemeters (Luminess badge). Hence, it was necessary to collect these data again. A dosemeter response experiment was conducted using a device that irradiated an anthropomorphic phantom in the Japan Atomic Energy Agency calibration laboratories. The aim of the paper is to provide a conversion factor from reading in terms of *H*_p_(10) to air kerma for realistic conditions for further conversion from air kerma to organ-absorbed dose. The obtained dosemeter responses for the dosemeter types currently used in Japan were consistent with those in the IARC study. These data will be utilized for J-EPISODE in reconstructing the organ-absorbed dose.

## INTRODUCTION AND AIM

The Japanese Epidemiological Study on Low-Dose Radiation Effects (J-EPISODE)^(1)^ has been conducted since 1990 and has analyzed the health effects associated with radiation exposure evaluated as the personal dose equivalent, *H*_p_(10). However, the evaluation of cancer morbidity and mortality using the organ-absorbed dose (Gy) is recommended by the International Commission on Radiological Protection (ICRP)^(2)^, and it was adopted for the 15-Country Collaborative Study of cancer risk among radiation workers in the nuclear industry conducted by IARC^(3–5)^, the International Nuclear Workers Study (INWORKS)^(6–9)^, Mayak study^(10)^ and the Life Span Study of atomic bomb survivors^(11–13)^. In addition, the incidence data by cancer site from the Japanese National Cancer Registry^(14)^, which became available in 2019, are indispensable for morbidity risk analysis with the organ-absorbed dose for the J-EPISODE.

The most comprehensive previous study that reconstructed the organ-absorbed dose from the recorded dose was set up in the framework of the IARC 15-Country Collaborative Study^(3,15)^, where experiments on dosemeter responses to photon exposure were performed for three types of dosemeters: the old film badge (FB), a multi-element FB and a thermoluminescence dosemeter (TLD). These types of dosemeters were used in the facilities that had participated in the IARC study from the inception of the nuclear industry until approximately 2000. These response data were useful for our study but were not sufficient, as those dosemeters have been replaced since 2000 by active personal dosemeters (hereafter called electronic personal dosemeters [EPDs]), radio-photoluminescent glass dosemeters (glass badges [GBs]) and optically stimulated luminescence dosemeters (Luminess badges [LBs]). In fact, until 2000, the dosemeters used in Japan were almost the same as those in the IARC study, so IARC study data could be used as they were. However, since 2000, the type of dosemeter has been changed, and it was necessary to collect those data again.

**Table 1:** The dosemeters selected for the study.

Type of dosemeter	Remarks
EPD	The Fuji Electric Co., Ltd. EPD-type NRG10811, which was in use at the Tokai Reprocessing Plant, JAEA, and calibrated in 2018. The measurement minimum unit: 0.01 mSv. Complied with JIS Z 4312: 2013^(16)^ based on IEC 61526: 2010^(17)^
GB^(18)^	The Chiyoda Technol Co. GBs (monitor code: FS) for wide-range Xγ and β, on the basis of radiophotoluminescence phenomena. Case type G-5 with plastic clip. The measurement minimum unit: 0.01 mSv.^a^ Complied with JIS Z 4345: 2017^(19)^ based on IEC 62387: 2012^(20)^
LB^(21)^	The Nagase-Landaure, Ltd. optically stimulated luminescence dosemeters, called ‘LB SG type’ for wide-range Xγ and β radiation. For the body trunk, with a plastic clip. The measurement minimum unit: 0.01 mSv.^a^ Complied with JIS Z 4345: 2017^(19)^ based on IEC 62387: 2012^(20)^

^a^While readings were rounded to one decimal place in the reports on measurement, the reference values with two digits after the decimal point were used for the study.

Investigation of the dosemeter response in a working environment requires a prior determination of the energy and geometry distribution of photon exposure. The IARC study estimated the organ dose conversion factor using the assumed photon energy distribution and geometry distribution, based on the judgement of experts at nuclear facilities around the world. Taking into consideration this assumption, the dosemeter response data under combinations of a specific photon energy: N-150 (mean energy: 119 keV), N-250 (207 keV) and ^137^Cs (662 keV), and a specific geometry (antero-posterior [AP] geometry and isotropic [ISO] geometry) were determined in the present study in the same way as they had been in the IARC study, but for an EPD, GB and LB. These data were then used to calculate the results for the personal dosemeter response in a working environment with an average photon energy distribution and geometry distribution experienced by Japanese nuclear workers.

The aim of this paper was to describe the dosemeter response determined by experiments conducted by the Radiation Effects Association at the calibration laboratories of the Japan Atomic Energy Agency (JAEA). The experiments examined three types of dosemeter: EPD, GB and LB in the same way of the IARC study. The organ-absorbed dose for nuclear industry workers was reconstructed from information obtained about the response of each dosemeter to photon radiation under a combination of energy ranges (100–300 keV and 300–3000 keV) and geometries (AP and ISO), where exposure in the rotational (ROT) geometry was considered negligible.

## MATERIALS AND METHODS

The method of the study followed the IARC study method. The goal was not to obtain a dosemeter response in a lab but to obtain it in an actual workplace. The response of personal dosemeter to AP, ROT and ISO exposures on the human body was compared with the dose delivered by calculation simulation. For this reason, an experiment was performed using a device that irradiated an anthropomorphic phantom while rotating it to simulate ROT and ISO.

### Dosemeters

For each type of dosemeter—EPD, GB and LB—a specific dosemeter was selected for the study, as listed in Table 1^(16–21)^. They all complied with Japanese standards^(16,19)^ based on international standards^(17,20)^, indicating that the type test was completed. Although there is no performance specification for discrete incident angles over 75°, it is unnecessary in phantom-rotating irradiation experiments, as mentioned before.

### Irradiation apparatus

Experiments were carried out at two calibration laboratories: the Facility of Radiation Standards (FRS) and the Instrument Calibration Facility (ICF) in JAEA, as described in detail elsewhere^(22,23)^. The FRS had ISO (International Standards Organization) X-ray narrow spectra series N-150 (tube voltage 150 kV, tube current 25.0 mA and mean energy 119 keV) and N-250 (250 kV, 15.5 mA and 207 keV)^(24)^ and the ICF had a ^137^Cs source with nominal radioactivity of 1.85 TBq.

### Simulation of working conditions

In the nuclear facilities under study, the workers would have been exposed to different irradiation geometries. The IARC study categorized facilities into two types: nuclear power plants (NPPs) and mixed activities (MA) facilities. The distribution of photon energy and geometry exposed in the working environment were then estimated as presented in Table 2^(3,15,25)^.

**Table 2:** Estimated percentage of the average dose from different photon energies and different geometries of exposure by nuclear facility type.

Nuclear facility type	Percentage of dose received from different energy photons (keV)			Percentage of dose received in different geometry		
	0–100	100–300	300–3000	AP	Isotropic	Rotational
NPPs						
Average dose (%)	0	10	90	50	50	0
MA facilities						
Average dose (%)	0	20	80	50	50	0

Reference: Table 4 of Thierry-Chef^(3)^.

**Figure 1:**
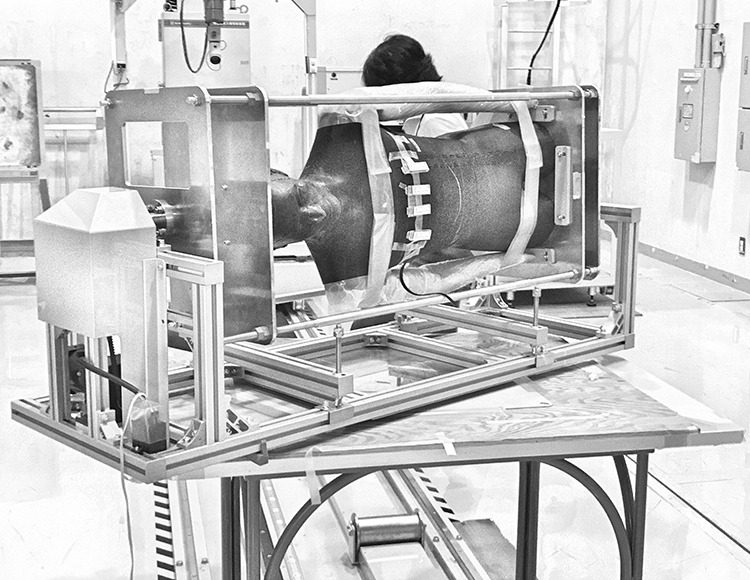
Device to rotate the RANDO phantom. The motor and gear case on the left. The turntable is located between the table and the device.

On average, in the NPPs, 10% of the dose was thought to be due to photon energies ranging from 100 to 300 keV and 90% from photon energies ranging from 300 to 3000 keV, with the average geometry being 50% in the AP geometry and 50% in the ISO geometry. Exposures in rotational geometry were considered negligible.

The results for the MA facilities indicated that 20% of the average dose was from photon energies ranging from 100 to 300 keV and 80% from photon energies ranging from 300 to 3000 keV. The predominant geometry of exposure was 50% in AP geometry and 50% in ISO geometry, on average.

### Phantoms and supporting device

The dosemeter response had to be assessed for both AP and ISO geometries. The body of the worker was simulated using two types of phantom. One was the slab phantom, which is a reference widely used for dosemeter calibration. It consisted of a water tank with polymethyl methacrylate walls (outer dimensions: 30 × 30 × 15 cm in depth).

The other was the anthropomorphic Alderson RANDO phantom^(26)^. Both arms of the CIRS ATOM^®^ phantom were attached, because an anthropomorphic phantom with arms was consistent with the phantom model used for computer simulation in ICRP Publ. 74^(27)^ or ICRP Publ. 116^(28)^. In addition, the effect of having both arms attached was verified by conducting a response test using the phantom with both arms removed.

A supporting device was constructed to rotate the anthropomorphic phantom in a horizontal position, around the body axis at a constant speed (45 s per rotation), as described by Tsujimura (2016)^(23)^. This device was fixed on a turntable and allowed the experimenter to change the angle between the source and rotation axis (Figure 1). The rotation center of the turntable was at the position of the 16th slice of the RANDO phantom; that is, the beam central axis from the source and the phantom rotation axis always intersected at the position of the 16th slice.

### Definition of dosemeter response

**Figure 2:**
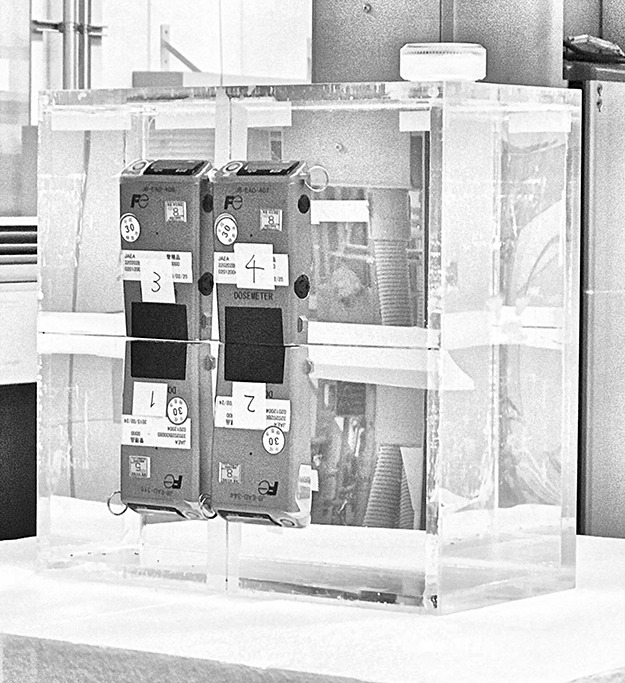
Position of the EPDs on the slab phantom.

**Figure 3:**
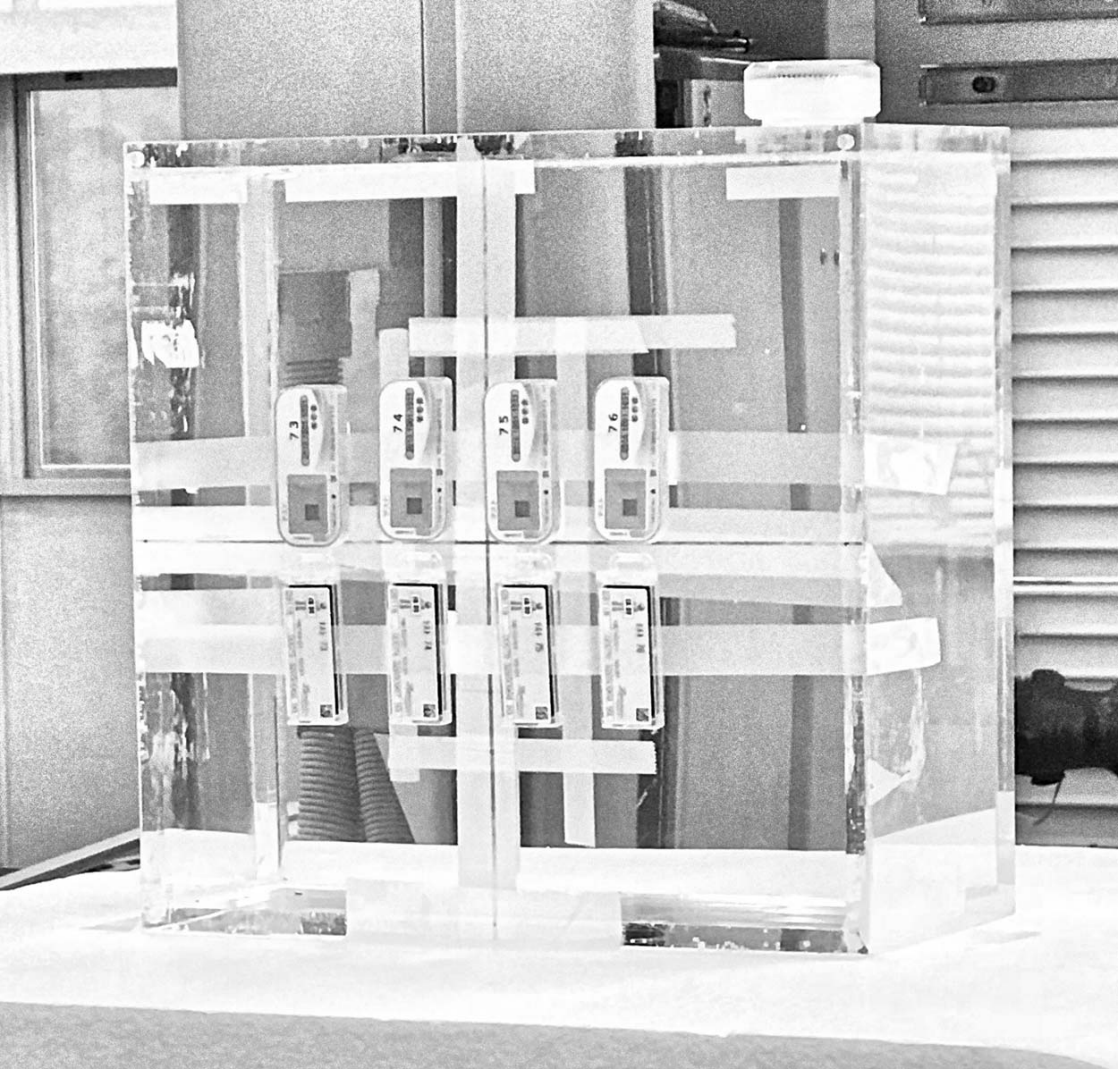
Position of the GBs (upper) and LBs (lower) on the slab phantom.

Before the RANDO phantom experiment, it was confirmed that the dosemeters used had displayed a response of 1.21 mSv per mGy under the standard calibration condition with the ^137^Cs source at 662 keV in the AP geometry. A calibration factor was obtained to correct individual differences in the sensitivities of dosemeters and systematic deviation of calibration among different dosemeters. The water slab surface was placed 3.5 m from the center of the reference source, and four personal dosemeters were taped around the center of the slab surface (Figures 2 and 3). For the EPDs, calibration factors were obtained for each dosemeter by placing and testing four EPDs in the experiment. For the GBs and LBs, which are passive dosemeters and whose quality of the element per lot was considered uniform, four samples from the same lot were tested, and the average of the readings were used for the computation of the calibration factor.

The calibration factor (mSv/mSv) was defined as an inverse ratio between reading and the reference *H*_p_(10) of 2.54 mSv. The dose rate of the air kerma on the surface of the water phantom was 10.5 mGy per h, the irradiation time was 720 s and the reference irradiation dose was 2.10 mGy. It was converted to *H*_p_(10) of 2.54 mSv using the conversion coefficient of 1.21 mSv per mGy, *H*_p_(10) per air kerma, which was interpolated from Table A.24 in ICRP Publ. 74^(27)^.

The dosemeter response (mSv/mGy) was defined by the following:

$$\begin{eqnarray*} &&\mathrm{Reading}\;\left(\mathrm{mSv}\right)\times \mathrm{Calibration}\kern0.17em \mathrm{factor} \;\left(\mathrm{mSv}/\mathrm{mSv}\right)/\\[-3pt]&&\qquad\qquad\mathrm{Reference}\kern0.17em \mathrm{dose}\;\left(\mathrm{mGy}\right) \end{eqnarray*}$$

Adequate precision was ensured by simultaneously irradiating four dosemeters of each type on the phantom. The average of the dosemeter response obtained for the reading of each dosemeter was taken as the average dosemeter response under each irradiation condition.

### AP geometry on the anthropomorphic phantom

The supporting device was installed so that the phantom rotation axis was 3.5 m from the center of the source. For AP irradiation, the rotation device was stationary, with the phantom facing straight toward the source. The dose of 3.38 m at the position of the phantom surface was used as a reference air kerma.

**Figure 4:**
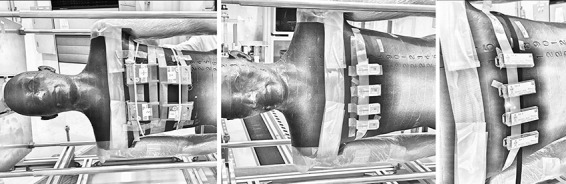
Position of the dosemeters on the RANDO phantom (EPDs, GBs and LBs, from left to right).

For each type of dosemeter and each source, the four holders were attached to the phantom and irradiated at a fixed angle of 90° to the beam central axis. On the RANDO phantom, dosemeters were placed on the 16th slice of the phantom, which corresponded to the position of the left pocket of the work clothing usually worn in most nuclear facilities in the study. Since the EPD had a large package case, two of the four EPDs were placed 15 cm apart at the position of the 16th slice, and the other two EPDs were placed upside down (Figure 4). The exposure dose for each irradiation test was evaluated by calculating the increment dose value from the readings each time.

### Isotropic geometry on the anthropomorphic phantom

**Figure 5:**
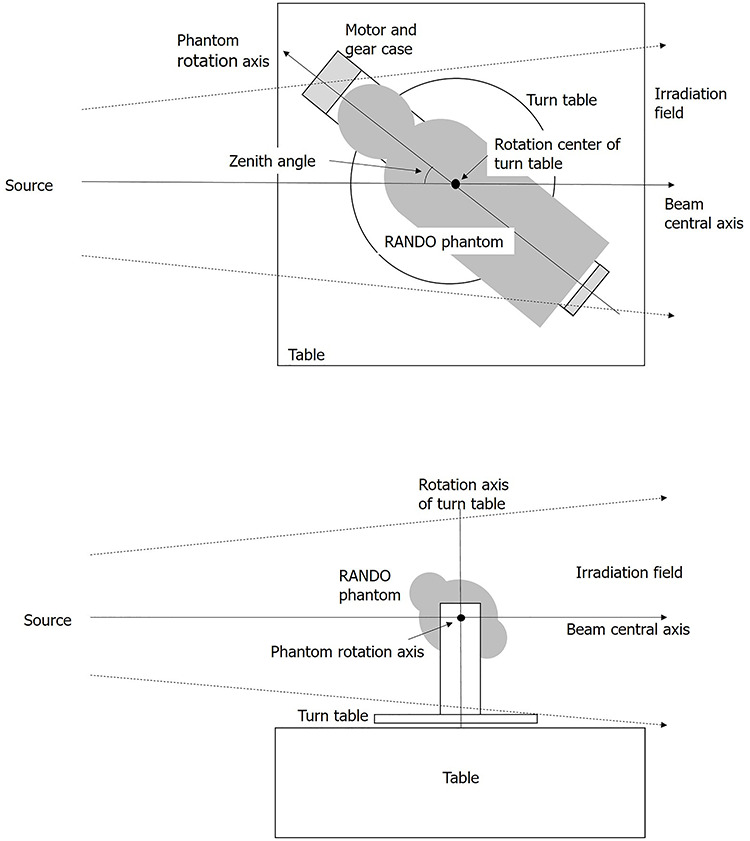
Simulation of isotropic irradiation.

The large proportion of the dose exposure in isotropic (ISO) geometry, as estimated by the IARC study, required that the dosemeter response be assessed in ISO geometry. The ISO geometry was simulated by rotating the phantom and changing the angle between the rotation axis and the source (Figure 5).

Four angles (zenith angles 30, 60, 120 and 150°) were studied to simulate ISO irradiation by combining the results obtained from the rotational (ROT) geometry of the exposure (zenith angle 90°).

A zenith angle of the 30° represents the weighted average of the area limited by 0 and 45°, the 60° angle was the weighted average of the area limited by 45 and 75° and the 90° angle was the weighted average of the area limited by 75 and 105°. Similarly, the 120 and 150° angles were the weighted average of the areas between 105 and 180°.

The response of the ISO geometry of exposure was obtained by integrating the results for rotation at each angle (integrating the differential cross section over the solid angle). The weight for each result was determined by the corresponding area. The response of the ISO geometry of exposure *B*_ISO_ was given by

$$\begin{equation*} {\displaystyle \begin{array}{l}{B}_{\mathrm{ISO}}=0.146\times{B}_{\mathrm{ROT}(30{}^{\circ})}\\ \kern2.1pc+0.224\times{B}_{\mathrm{ROT}(60{}^{\circ})} + 0.259\times{B}_{\mathrm{ROT}(90{}^{\circ})}\\ \kern2.2pc+0.224\times{B}_{\mathrm{ROT}(120{}^{\circ})}+0.146\times{B}_{\mathrm{ROT}(150{}^{\circ})}\end{array}} \end{equation*}$$

with *B*_ROT(*θ*)_ being the response at zenith angle of *θ*.

For each source and each angle, the dosemeters were irradiated during an integer number of rotations. This number was determined based on the motor speed and the duration required to obtain an approximately 1-mGy air kerma from each source, as shown in Table 3.

The dose at the 3.5-m phantom rotation axis was taken as the reference air kerma. The supporting device was fixed at 3.5 m from the source for the rotation axis. Because rotation was performed at angles toward the source, the distance between the source and the dosemeter was not exactly 3.5 m during the rotation. However, since the number of rotations was an integer, this resulted in an equal opportunity for the distance to become longer than 3.5 m and to become shorter, indicating no problem in applying the dose rate of 3.5 m.

### Measurement errors

In addition to the mean value of the dosemeter response, the standard deviation (SD) was calculated to estimate the uncertainty *K* under the assumption of lognormal distribution for the variable of dosemeter response *B* for each type of dosemeter, each category and each dosimetry.

$$K=\exp\;\left(1.96\times \mathrm{SD}\right)$$

## RESULTS

### Calibration test before the RANDO phantom experiment

The calibration factor obtained with the ^137^Cs source at 662 keV in AP irradiation was calculated as the ratio between the reference *H*_p_(10) dose of 2.54 mSv and the dosemeter reading. The individual dosemeter readings of the subsequent irradiation test were multiplied by the calibration factors. The value of the calibration factors was 1.01–1.04, that is almost equal to unity. It was confirmed that the dosemeters used almost displayed 1.21 mSv per mGy under the standard calibration condition.

### Mean response on the RANDO phantom in specific conditions

Table 4 shows the mean response and uncertainty for each type of dosemeter irradiated on the RANDO phantom at each of the three energies and three geometries of exposure.

In summary, the dosemeter responses shown in Table 4 according to dosemeter type (EPD, GB and LB), energy and geometry indicate the following.

(1) For AP irradiation at 662 keV, the response was about 1.2 mSv per mGy for all dosemeter types, showing that the response of AP geometry on the RANDO phantom was not much different from AP geometry on the slab phantom, which was used practically for calibration. A lower energy gave a larger response value. However, in comparison with the values of *H*_p_(10, 0°)/*K*_a_ interpolated from Table A.24 in ICRP Publ. 74^(27)^, the relative response to *H*_p_(10) per air kerma at 119 keV was 0.79 for EPD, 0.84 for GB and 1.13 for LB, which were within the range allowed by the standards.
(2) The ISO response value was about 0.8–0.9 mSv per mGy at 662 keV (EPD 0.82, GB 0.86 and LB 0.89). The difference in response due to energy was smaller for the ISO geometry than for the AP geometry. For instance, the value of response for LB at 119 keV in ISO geometry was 1.14 mSv per mGy, which is 28% larger than the value of 0.89 at 662 keV, while the response value of 1.93 for LB at 119 keV in AP geometry was 66% larger than the value of 1.19 at 662 keV.
(3) The response values were smaller for ISO than for AP for all dosemeter types and energies. In particular, a lower energy gave a larger difference for the AP. At 119 keV, the response values in ISO (EPD 0.77, GB 0.79 and LB 1.14) were smaller than those in AP; EPD was 44% smaller, GB 46% and LB 42%, respectively.
(4) For LB at 662 keV, the response value of 0.96 for ROT (90°) was larger than that for the oblique geometry ROT (*θ*); it was 0.88 for ROT (60°) and 0.87 for ROT (120°). The EPD and GB did not show such tendencies.
(5) The response values for ROT (*θ*) showed no major differences for *θ* = 30–120° regardless of the energy. For instance, the value of ROT (*θ*) was 0.87–0.89 mSv per mGy for GB when *θ* being between 30 and 120°, but it decreased to 0.79 when *θ* was 150°. The response of ROT (150°) might be affected by the shielding effect of the waist.

### Mean response in a working environment

The personal dosemeter response in a working environment with the average photon energy distribution and geometry distribution experienced by Japanese nuclear workers is shown in Table 5. According to the IARC study^(3)^, the dosemeter response in the 100–300 keV range was considered to be represented by the response at 119 and 207 keV and was computed as the weighted average (each ratio 25:75%) of each result. In addition, the dosemeter response in the 300–3000 keV range was considered to be represented by a point at 662 keV.

**Table 3:** Irradiation conditions of the dosemeter response experiments by energy and geometry.

Narrow beam series/quality of dose		N-150	N-250	^137^Cs
Mean energy measured		119 keV	207 keV	662 keV
Dose rate of air kerma	AP (3.38 m)	27.6 mGy per h	7.88 mGy per h	11.3 mGy per h
	ROT (3.5 m)	25.7 mGy per h	7.33 mGy per h	10.5 mGy per h
Duration	AP	315 s	990 s	720 s
	ROT	180 s	445 s	360 s
		(4 rotations)	(11 rotations)	(8 rotations)
Reference air kerma	AP (3.38 m)	2.41 mGy	2.17 mGy	2.26 mGy
	ROT (3.5 m)	1.28 mGy	0.91 mGy	1.05 mGy

ROT: Rotational geometry

**Table 4:** Response of dosemeters irradiated on a phantom at three radiation energies (119, 208 and 662 keV) in AP and isotropic geometries of exposure.

Dosemeter type	Geometry	Mean response (*B*) (mSv/mGy)			Uncertainty (*K*)		
EPD		119 keV	207 keV	662 keV	119 keV	207 keV	662 keV
	AP	1.38	1.35	1.20	1.010	1.008	1.000
	ROT (30°)	0.84	0.99	0.87	1.021	1.008	1.031
	ROT (60°)	0.83	0.97	0.86	1.021	1.027	1.008
	ROT (90°)	0.83	0.97	0.87	1.011	1.015	1.014
	ROT (120°)	0.78	0.92	0.83	1.018	1.029	1.015
	ROT (150°)	0.51	0.69	0.64	1.039	1.041	1.022
	ISO	0.77	0.92	0.82	1.009	1.011	1.008
GB		119 keV	207 keV	662 keV	119 keV	207 keV	662 keV
	AP	1.47	1.30	1.21	1.046	1.035	1.021
	ROT (30°)	0.65	0.84	0.87	1.056	1.059	1.072
	ROT (60°)	0.80	0.89	0.87	1.014	1.037	1.036
	ROT (90°)	0.83	0.92	0.88	1.040	1.017	1.015
	ROT (120°)	0.82	0.90	0.89	1.059	1.021	1.009
	ROT (150°)	0.80	0.79	0.79	1.058	1.023	1.020
	ISO	0.79	0.88	0.86	1.020	1.014	1.014
LB		119 keV	207 keV	662 keV	119 keV	207 keV	662 keV
	AP	1.98	1.55	1.19	1.049	1.062	1.079
	ROT (30°)	1.15	1.01	0.90	1.055	1.065	1.097
	ROT (60°)	1.14	1.03	0.88	1.127	1.064	1.066
	ROT (90°)	1.17	1.08	0.96	1.046	1.017	1.060
	ROT (120°)	1.16	1.07	0.87	1.042	1.083	1.034
	ROT (150°)	1.09	0.90	0.80	1.128	1.021	1.094
	ISO	1.14	1.03	0.89	1.037	1.025	1.029

AP: antero-posterior geometry; ISO: isotropic geometry; ROT: rotational geometry.

Notes: (1) Uncertainty *K* of the dosemeter response at a certain energy and geometry category was obtained by the following equation, where SD is the standard deviation of the logarithm of each response estimate.

*K* = exp (1.96 × SD)

Hence, the 95% confidence interval of the mean response *B* was expressed as (*B*/*K*, *B* × *K*) using the uncertainty of *K*.

(2) *K*_ISO_, uncertainty in ISO geometry, was defined as follows using *K*_ROT(*θ*)_, the uncertainty of the dosemeter response of each ROT (*θ*), as well as the corresponding weight.

*K*_ISO_ = exp(1.96 × sqrt(0.146^2^ × *K*_ROT(30°)_^2^ + 0.224^2^ × *K*_ROT(60°)_^2^ + 0.259^2^ × *K*_ROT(90°)_^2^+ 0.224^2^ × *K*_ROT(120°)_^2^ + 0.146^2^ × *K*_ROT(150°)_^2^)).

(3) The results for the EPD were obtained from only two dosemeters placed at the position of the 16th slice in the present study. The gap of evaluation values between these two EPDs and the other two diverged more than 30% at the zenith angles of 30 and 150° in the energy 100–300 keV due to the differences in the attachment position and the inclination of phantom surface. The EPD used in the experiment had a battery at the bottom, which might have yielded a smaller response to the beam when irradiated from below.

According to Table 5, the mean dosemeter response (mSv/mGy) in the working environment was close to 1 for both the NPP and the MA facility for each type of dosemeter.

### Measurement error in dosimetry

For the EPD, GB and LB, the minimum unit of measurement was 0.01 mSv, and the rounding error was 0.005 mSv. The measurement error for a dosemeter was defined as the SD of the individual deviation rates from the mean readings. This value was 1.1% for the EPD, 2.0% for the GB and 3.5% for the LB.

The error was considered to arise due to the measurement error of the dosemeter and due to the measurement method, such as the differences in the inclination of the phantom surface depending on the dosemeter attachment position and the differences in distance between the phantom rotation axis and the dosemeter.

No effect was noted for background radiation. The evaluation value for the personal dosemeter used as a control to confirm background radiation was below the detection limit for the GB and 0 mSv for the LB.

**Table 5:** Dosemeter response and uncertainty by dosemeter type and nuclear facility type.

Dosemeter type	Response (*B*) (mSv per mGy)		Uncertainty (*K*)	
	NPP	MA	NPP	MA
EPD	1.00	1.01	1.004	1.003
GB	1.02	1.02	1.011	1.011
LB	1.06	1.08	1.037	1.033

Notes: Dosemeter response *B* and uncertainty *K* by facility type were estimated using the values of *B* and *K* determined by the energy in the AP and isotropic (ISO) geometry in Table 4, as well as the corresponding weights.

## DISCUSSION

### Differences from IARC study

The experiment on the dosemeter response was conducted essentially as described by the IARC study^(3,15)^. Some details differed as shown in Table 6, but they did not affect the comparability of the experimental results.

**Table 6:** Differences in the experiments between the present study and the IARC study.

	The present study	The IARC study
Type of dosemeter	EPD, GB and LB	Old FB, Multi-element FB and TLD
Common quantity	Air kerma	*H*_p_(10)
Definition and unit of dosemeter response	*H*_p_(10)/air kerma; mSv/mGy;	*H*_p_(10) assessed/*H*_p_(10) delivered; mSv/mSv
Distance between the source and the phantom	3.5 m	2.0 m
Reference air kerma delivered in ROT (*θ*) geometry	About 1 mGy	About 5 mGy
Arms of RANDO phantom	Attached	Not attached

Since the IARC study included the old type of dosemeters used in the 1940s or 1950s, the *H*_p_(10) was adopted as a common quantity. For this reason, *H*_p_(10) assessed/*H*_p_(10) delivered (mSv/mSv) was used as a definition of the dosemeter response to correspond to various dose concepts and units.

The distance from the source, the reference dose in ROT geometry and the difference with/without both arms of the phantom had no effect on the dose response value measured in the assumed working environment.

The distance between the source and the phantom was set to 2 m in the IARC study, but it was set to 3.5 m in the present study so that the whole phantom was included in the irradiation field, taking into account the opening angle of the conical collimator of the X-ray irradiator and ^137^Cs gamma-ray irradiator.

The AP geometry defined by ICRP assumes an ideal field of exposure to a parallel beam from a planar source. Conversely, the irradiation apparatus used in the experiment was a divergent radiation beam from a point source. When comparing the responses measured in the ROT geometry at different energies (119, 207 and 662 keV) and the distances from the source (2, 3, 4 and 5 m) to those calculated with collimated beams, the degree of deviation was larger at the lower energy and at the shorter distance. At a distance of 3.5 m, the deviation was 1–2% at 662 keV and 2–3% at 119 keV, revealing a negligible difference when compared with the parallel beam response.

We irradiated the dosemeters with 1 mGy in the ROT geometry, whereas IARC study irradiated with 5 mGy. In working conditions, exposures would be far below these levels. These irradiations were considered sufficient to obtain accurate results and to minimize the uncertainty.

In the experiment, the dosemeter response test was conducted with both arms attached to the RANDO phantom. Comparison of the dosemeter response in the presence and absence of both arms under the same irradiation condition (662 keV in ROT [90°] geometry, for the EPD) showed that the total response was 0.83 mSv per mGy with both arms and 0.84 without both arms. Although slight, a shielding effect by wearing both arms was recognized.

#### Limitations of the experiment

The personal dosemeters investigated in this study have been used in Japan since around 2000 and were not covered in the IARC study. The original type of dosemeter, which is supposedly representative of those used in the period of interest, is no longer produced, so the type in current use was studied in this experiment. However, as seen in Table 5, the difference in dosemeter response between dosemeter types is small, so dosemeter selection for the experiment did not appear to affect the mean dosemeter response in the assumed working environment.

#### Data from IARC study

The IARC study examined 10 historical dosemeters. The experimental results were described in Table 3 of Thierry-Chef^(15)^, as well as in the Web annex Table 4 of Thierry-Chef^(3)^. We referred to the first table because it had more significant digits. In order to compare the results of the IARC dosemeter responses and those of the present study, the values were converted to dosemeter response per air kerma. In the IARC study, the values in Table 3^(15)^ were obtained from ‘readings per air kerma’ divided by ‘conversion coefficient from air kerma to *H*_p_(10)’ from Table 1^(15)^, as the following:

$$\begin{eqnarray*} \left[\mathrm{Readings}/\mathrm{air}\;\mathrm{kerma}\right] /\left[{H}_{\mathrm{p}}(10)/\mathrm{air}\;\mathrm{kerma}\right]=\left[\mathrm{Readings}/{H}_{\mathrm{p}}(10)\right] \end{eqnarray*}$$

Therefore, the values in Table 3^(15)^ could be converted to dosemeter response per air kerma by multiplying by the ‘conversion coefficient from air kerma to *H*_p_(10)’ from Table 1^(15)^.

The X-ray quality and beam code of the IARC study were the same as in our study: ISO 4037-1/N-150 and N-250^(24)^, with a mean energy of 118 and 208 keV, respectively, and the differences of the mean energy were negligible.

The dosemeter types used in Japan before 2000 were roughly categorized into three types in IARC study as the old FB, multi-element FB and TLD. Reconstruction of the organ-absorbed dose in the J-EPISODE study required selection of the most suitable of the 10 dosemeter types used in the IARC study.

### FBs previously used in Japan

We classified the FBs used in Japan into two types. The first type was the JIS II type FB for gamma-ray based on JIS Z 4302 (1957)^(29)^, one of the oldest FB used in Japan, mainly in research organizations. This type was regarded as an old FB in the IARC study. The average dosemeter response of FR-1, US-2, UK-2 and UK-5 was assigned to the old FB in Japan.

The other FB type was the multi-element FB of the IARC study and corresponded to the average of the UK-9, US-8 and FR-6 (Table 7).

**Figure 6:**
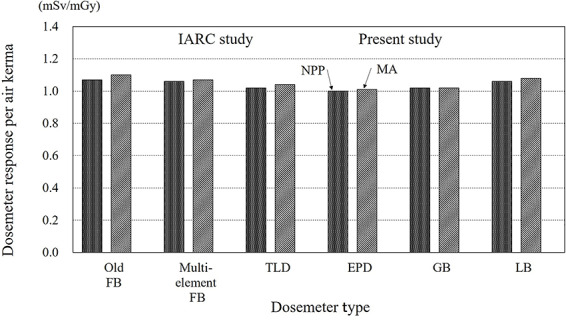
Dosemeter response per air kerma in the work environment experienced by nuclear workers by dosemeter type and nuclear facility type.

**Table 7:** Dosemeter types used in Japan and the corresponding data from the IARC study.

Dosemeter types used in Japan		Corresponding data for the dosemeter response in the IARC study
Old FB	JIS II type FB for γ-ray	Average of FR-1, US-2, UK-2 and UK-5
Multi-element FB	Other FBs	Average of UK-9, US-8, and FR-6
TLD	Panasonic product	US-22

### TLDs used in Japan

A rigorous comparison of the TLD badges used in Japan with those used in the IARC study was difficult in terms of TLD materials and packaging cases. This was because the TLD badge used in Japan was a Panasonic UD-808 consisting of a resin filter, equivalent to 1000 mg per cm^2^, that covered a Li_2_B_4_O_7_(Cu) element. In the IARC study, the UK-10 and FR-9 were selected as the LiF-based TLDs dominant in the West, while the US-22 (Panasonic UD-802) was selected as a combination of a CaSO_4_(Tm) element and a lead filter. Strictly speaking, although US-22 made by Panasonic used at the Savannah River Site in the USA differed slightly from the model numbers and filters of the TLDs used in Japan, the similarity in the basic dosemeter structure indicated that the data for the US-22 were most suitable for comparison with the TLD used in Japan.

### Dosemeter response by dosemeter type used in Japan

Figure 6 shows the dosemeter responses from the IARC study and the present study, and the results are consistent. The results of the three types on the left in Figure 6 are from the IARC study, and those on the right are the experimental results from the present study.

### Applicability of the IARC study assumption to Japanese nuclear workers

The assumption of the IARC study on the energy and geometry distribution of photons is fundamental information in estimating organ-absorbed dose from the reading of personal dosemeters. The results of the literature survey in the 1980s found that there were investigations for which electric power companies actually measured energy and geometry distributions at NPPs. The evidence of the working environments of Japanese workers in NPP demonstrated the appropriateness of applying the IARC study assumption to reconstructing organ-absorbed dose in J-EPISODE, as described elsewhere^(30)^.

## CONCLUSIONS

The data for the dosemeter response in a working environment are fundamental information when reconstructing the organ-absorbed dose. The dosemeter response experiment conducted in the present study for EPD, GB and LB dosemeters was intended to rectify the lack of response data for these dosemeters. The dosemeter response data obtained for the dosemeter types in current use in Japan were consistent with those reported in the IARC study for the old FB, multi-element FB and TLD. These data will be utilized for J-EPISODE in reconstructing organ-absorbed doses. The findings will also be useful for any nuclear workers cohorts using the new types of dosemeters.
